# The effect of testosterone replacement therapy on bladder functions, histology, apoptosis, and Rho-kinase expression in bladder outlet obstruction and hypogonadism rat model

**DOI:** 10.3906/sag-2004-15

**Published:** 2021-06-28

**Authors:** Barış SAYLAM, Ozan EFESOY, Rabia BOZDOĞAN ARPACI, Rukiye Nalan TİFTİK, Mesut TEK, Kansu BÜYÜKAFŞAR, Murat BOZLU, Selahittin ÇAYAN

**Affiliations:** 1 Department of Urology, Mersin City Training and Research Hospital, Mersin Turkey; 2 Department of Pathology, Faculty of Medicine, Mersin University, Mersin Turkey; 3 Department of Pharmacology, Faculty of Medicine, Mersin University, Mersin Turkey; 4 Department of Urology, Faculty of Medicine, Mersin University, Mersin Turkey

**Keywords:** Bladder, rho-kinase expression, hypogonadism, apoptosis, bladder outlet obstruction

## Abstract

**Background/aim:**

The effect of testosterone replacement therapy was investigated on bladder functions, histology, apoptosis as well as Rho-kinase expression in the rat bladder outlet obstruction (BOO) and hypogonadism models.

**Materials and methods:**

30 mature male rats divided into 4 groups: sham group (n = 8), BOO group (n = 8), BOO + orchiectomy group (n = 7), BOO + orchiectomy + testosterone (T) treatment group (n = 7). Cystometric findings, apoptosis index, Rho-kinase (ROCK-2) expression, and smooth muscle/collagen ratio were compared.

**Results:**

BOO did not change ROCK-2 expression level, compared to sham group (P > 0.05). However, when compared to BOO group (P < 0.01), BOO + orchiectomy led ROCK-2 increase. The testosterone treatment failed to reverse the up-regulation of ROCK-2 induced by orchiectomy although it tended to lower ROCK-2 level. Compared to sham group (P = 0.002), changes in maximal bladder capacity and leak point pressure were higher (P = 0.026, P = 0.001), and bladder compliance was lower in BOO group. Also, the apoptosis index was different between the two groups (P = 0.380). Smooth muscle/collagen ratio was higher in BOO + orchiectomy + T group than in BOO + orchiectomy group (P = 0.010).

**Conclusions:**

The research draws attention to alternating treatment approaches in case of the presence of hypogonadism and BOO.

## 1. Introduction

Benign prostatic hyperplasia (BPH) is a disorder that can cause bladder outlet obstruction (BOO). BPH is the most important pathological condition that contributes to lower urinary tract symptoms (LUTS). LUTS increases with age and its prevalence above 50 years is approximately 50%. Two-thirds of men with BPH experience symptoms of overactive bladder (OAB). BOO causes detrusor smooth muscle (DSM) hypertrophy in the bladder, which leads to impaired bladder storage and voiding ability. After pharmacological or surgical treatment of BOO, OAB symptoms may still persist in 38% of men [1,2]. 

Smooth muscle structure and function changes significantly after BOO. Failure of the bladder to adapt to BOO is the main reason for reduced bladder contraction function [3]. Adequate smooth muscle and collagen content and their ratio in bladder interstitium are important for bladder function. Smooth muscle contractions of the bladder are regulated by intracellular Ca2+ concentration. Increased intracellular Ca2+ concentration causes calmodulin-mediated activation of myosin light chain kinase (MLCK), which causes smooth muscle contraction. In addition to the calcium-dependent pathway, the Rho-kinase pathway, a calcium-independent pathway, has gained importance in smooth muscle contraction [4]. Rho-kinase (ROCK) is a serine/threonine protein with two isoforms: ROCK α and ROCK β. It has been proven that the Rho-kinase pathway plays an important role in the regulation of bladder smooth muscle contraction and tone. Activated ROCK phosphorylates and deactivates smooth muscle myosin phosphatase, which prevents phosphorylation of myosin light chain kinase, which leads to Ca2+ sensitization of smooth muscle [5]. ROCK is one of the main mediators for muscle contraction and relaxation responses. The important role of ROCK protein in expression and smooth muscle cell contraction has been demonstrated in cavernous bodies, ureters, bladder, and vas deferens [6]. In BOO, detrusor muscle hypertrophy occurs, and ROCK expression increases [7]. 

Androgen deficiency is a condition that causes urogenital dysfunction in older men. In symptomatic late-onset hypogonadism (SLOH), testosterone replacement therapy can lead to prostate enlargement and consequently LUTS [8]. Testosterone replacement therapy is indicated in the presence of clinical symptoms suggestive of hormone deficiency and decreased testosterone level [9]. The aim of this study is to investigate the effect of Rho-kinase expression and testosterone replacement therapy on bladder functions, histology, apoptosis and Rho-kinase expression in BOO and hypogonadism rat model.

## 2. Materials and methods

The experimental protocol was approved by the ethical committee on Animal Research at the University of Mersin School of Medicine (Approval No: 200917). A total of 30 Wistar-albino rats were included in the study and divided into 4 groups. The rats weighed 200–250 g and were 30 days old. The sham group (n = 8) included sham operated rats; BOO was performed in the BOO group (n = 8) and BOO and bilateral orchiectomy in the BOO + orchiectomy group (n = 7). In the BOO + orchiectomy + T treatment group, rats were given intramuscular injection of 100 mg/kg testosterone undecanoate (Nebido, Bayer Schering Pharma, Berlin, Germany) after bladder outlet obstruction and bilateral orchiectomy (n = 7). In all groups, urodynamic evaluations were performed to determine bladder pressures, capacities and compliance at the beginning of the study and 2 months later. At the end of the study, after the rats were sacrified, smooth muscle collagen ratios and apoptosis indices were studied. Rho-kinase (ROCK-2) protein expression in bladder tissue was determined by Western blot analysis. 

### 2.1. Surgical model of bladder outlet obstruction

Under sterile conditions, moderate prostatic urethral obstruction was created in all rats by retropubic approach after anesthetic induction with ketamine (80–100 mg/kg i.p.). 150 mg/kg ampicillin i.m was given for prophylaxis before the procedure. The urinary bladder was reached with a 2 cm midline incision and the prostatic urethra was carefully isolated. To create a partial occlusion of the urethra, a 0.91 mm sterile metal rod was attached around the prostatic urethra with 3–0 polypropylene nonabsorbable suture, and when the suture was fixed, the metal rod was slowly removed. The upper muscle layers were brought closer with 4–0 silk suture. The skin was then closed, and the incision was cleaned with povidone-iodine. Rats were placed in a cage under a heating lamp after surgery.

### 2.2. Functional evaluation

Urodynamic studies were performed at the beginning and end of the study just before the rats were sacrified [10]. After the midline incision was made under ketamine anesthesia (50 mg/kg i.m.), the 22 - G catheter was placed directly in the dome of the bladder and the residual urine was emptied. The catheter was connected to a pressure transducer using urodynamic equipment with a polyethylene tube, and urodynamics were performed by infusing a heated (37 °C) normal saline solution at 0.15 mL/min. Urodynamic studies were performed at the beginning and end of the study in each rat. The baseline (empty bladder), opening (at the first leakage), and peak pressure (maximal pressure during voiding) and the maximal bladder capacity were recorded during the study. Bladder compliance (ml/cm H2O) was calculated according to the following formula: 

Compliance = Maximal Bladder Volume / Opening Pressure–Baseline Pressure.

### 2.3. Western blot analysis for ROCK-2

Rat bladder tissues were homogenized with a lysis buffer (composition in mM; Tris–HCl (pH = 7.4) 50 mM, NaCl 400 mM, EGTA 2 mM, EDTA 1 mM, dithiothreitol 1 mM, phenylmethylsulfonyl fluoride 10 μM, leupeptin 10 μg/ml, pepstatin 1 μg/ml, benzamidine 1 mM). The homogenate was centrifuged at 13,000 × g for 10 min at 4 °C, and the supernatant was removed. It was then used for protein analysis (with Bradford method) and Western blot analysis [11]. Equal amounts of the protein (75 μg) were loaded in wells, electrophoresed on 8% polyacrylamide–sodium dodecyl sulphate (SDS) gels and then transferred to a nitrocellulose membrane overnight. The membrane was blocked with the blocking agent of the enhanced chemiluminescence (ECL advance) kit (Amersham Biosciences, Freiburg, Germany) in Tris-buffered solution containing 0.05% Tween 20 for 1 h. It was then probed with a primary antibody raised against ROCK-2 (ROCKα, Mouse monoclonal IgG, BD Science, USA) at 1: 2000 dilution followed by a horseradish peroxidase (HRP) - conjugated secondary antibody (donkey antimouse, 1: 2000, Santa Cruz Biotechnology Inc. CA, USA). Protein blots were then detected with the advanced chemiluminescence detection kit (Amersham Biosciences, Freiburg, Germany) and visualized on commercial X-ray film. 

### 2.4. Histologic evaluation

**Figure 1:**
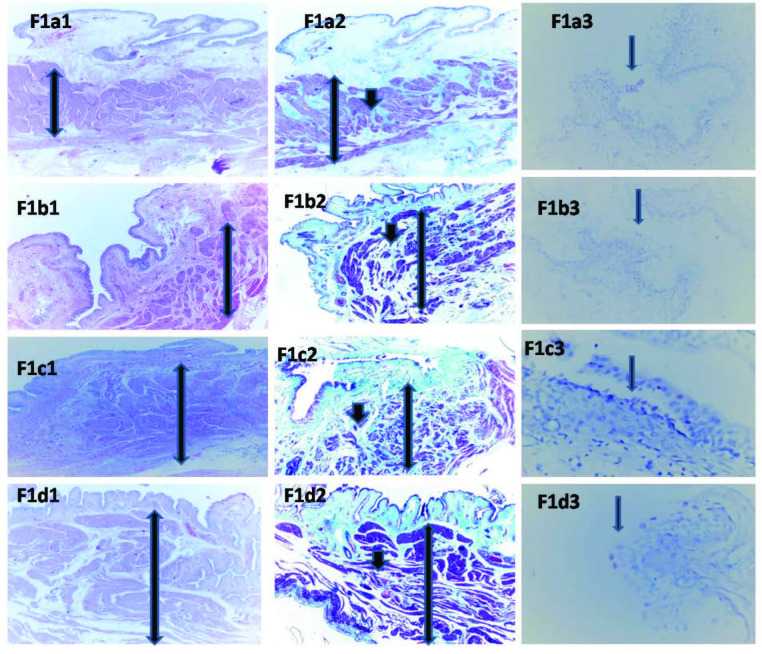
Histological sections shows a regular bladder muscle thickness in the sham group. A slightly increased ratio in favor of muscle is observed histologically (a1: H-E,  40). By the Masson’s trichrome stain, fibrous tissue (green and marked with a small arrow) and regular muscle tissue (red and marked with a long arrow) were seen (a2; Masson Trichrome,  40). The number of apoptotic cells per unit focus in the whole epithelial lining area were shown immunohistochemically. The determined apoptotic cells were 6–8 (arrow) in a high power field (HPF), microscopically. (a3: Tunnel,  400). 1b. Histological sections shows minimally increased bladder muscle in the BOO group. A slightly increased muscle fibers were observed (b1: H-E,  40). The minimally increased fibrous tissue (green and marked with a small arrow) around perifascicular and intrafascicular area and minimally increased muscle tissue (red and marked with a long arrow) were seen by Masson’s trichrome stain (b2; Masson Trichrome,  40). The determined apoptotic cells were 7–8 (arrow) in a high-power field (HPF) immunohistochemically (b3: Tunnel,  400). 1c. Histological picture shows a partially increased regular bladder muscle thickness in the BOO + orchiectomy group (c1; H-E,  40). With Masson’s trichrome stain, fibrous tissue (green and marked with a small arrow) is observed in muscle tissue (red and marked with a long arrow) in a slightly increased ratio (c2; Masson Trichrome, x40). The number of apoptotic cells (7-8/ HPF) (arrow) do not show a significant difference in the form of sham group (7-8/HPF) (c3: Tunnel,  400). 1d. The image shows a markedly thickened bladder muscle in the BOO + orchiectomy+ treatment group (d1; H-E,  40). When compared with Masson’s trichrome stain, especially in Figure 1a, fibrous tissue (in green color) is quite rare and a markedly increased irregular muscle fibers (in red color) were observed (d2; Masson Trichrome,  40). The number of apoptotic cells were observed by immunohistochemistry does not show a significant difference. Apoptotic epithelial cell count is 7–8/5 HPFs (arrow) (d3: Tunnel,  400).

In paraffin sections (5 μm), bladder tissues were placed and encoloured with Masson’s trichrome for the assessment of smooth muscle/collagen content. The sections were assessed using light microscopy at 400 hpf., and intended areas were photographed. The muscularis propria was encoloured red, and the collagen deposition of intrafascicular and perifascicular zone of the muscularis propria was encoloured using green light. The muscle ratio of the rats and the collagen tissue deposition were evaluated as 1unit fibrosis and 3units muscle: mild blockage (1), 2 unit fibrosis and 2 unit muscle: moderate blockage (2), and 3 unit fibrosis and 1 unit muscle: marked blockage (3). Histologic pictures of the bladder mucosa (Figure 1a1, Figure 1b1, Figure 1c1, and Figure 1d1), smooth muscle and collagen tissue deposition of all groups were shown in Figure 1a2, Figure 1b2, Figure 1c2, and Figure 1d2.

For detection of apoptosis, the terminal deoxynucleotidyl transferase-mediated dUTP-nick end labelling (TUNNEL) method was employed, histologic pictures of apoptotic cells were shown in Figure 1a3, Figure 1b3, Figure1c3, and Figure 1d3. In brief, 5 μm density of tissue sectors were deparaffinized and rehydrated in a graded series of xylene and ethanol. The staining progress was monitored with the help of a microscope right after the encolouring by 0.05% diaminobenzidine in staining buffer. The zone with the highest number of apoptotic cells was chosen at five high-power (× 400) branches. Next, 100 cells were regarded, and the apoptotic index was described as the ratio of the apoptotic cells to the normal epithelial cells in a dedicated zone.

### 2.5. Statistical analysis

Statistical analyses were performed using the “one way ANOVA test” to compare the mean body weight and mean serum testosterone level at the beginning and end of the experiment among the groups, “independent t–test” to compare smooth muscle collagen ratio and differences as percentage in cystometric findings at the beginning and end of each experiment between the 2 groups. Data are presented as a mean ± standard deviation for the smooth muscle/collagen ratio. Probability values of <0.05 were considered statistically significant. For Western-blot analysis, all data represent mean ± standard errors of the mean (S.E.M.) of n observations. Shapiro–Wilk as normality test was performed all parameters and comparing two or more groups. All parameters and groups were in normal distribution (P values >0.005, 95% confidence interval), and we used parametric tests. For statistical comparison, analysis of variance (ANOVA) followed by Bonferroni’s multiple comparison test was used. A P value of less than 0.05 was considered significant. Graphs were drawn by the use of a GraphPad Prism 3.0 program (GraphPad Software, San Diego, CA, USA).

## 3. Results

As shown in Table 1, there were no differences in the mean body weight and total testosterone level at baseline among the groups. From the beginning to the end of the experiment, no significant differences in the mean weight were seen in the BOO and BOO + orchiectomy groups; however, the mean body weight significantly increased in the sham and BOO + orchiectomy + T treatment groups (P < 0.001 and P < 0.001, respectively). The mean serum total testosterone level did not change in the sham, BOO and BOO + orchiectomy + T treatment groups; however, it significantly decreased in the BOO + orchiectomy group form the beginning to the end of the experiment (P = 0.001).

**Table 1:** Mean weight, total testosterone level, and cystometric findings (maximal bladder capasity, leak point pressure and compliance) of the rats from the beginning to the end of the experiment in all groups.

	Sham group (n = 8)	BOO group (n = 8)	BOO + orchiectomy group (n = 7)	BOO + orchiectomy + T treatment group (n = 7)
Basal weightLast weight (g)	233.88 ± 4.67264.50 ± 5.26(P < 0.001)	234.88 ± 5.19240.38 ± 8.67(P = 0.059)	230.14 ± 6.20229.14 ± 5.37(P = 0.582)	232.00 ± 5.13261.71 ± 7.78(P < 0.001)
Basal total testosteroneLast total testosterone (ng/mL)	0.507 ± 0.0420.503 ± 0.024(P = 0.676)	0.519 ± 0.034 0.509 ± 0.042 (P = 0.086)	0.505 ± 0.034 0.002 ± 0.001 (P = 0.001)	0.513 ± 0.021 0.514 ± 0.014 (P = 0.889)
Basal max. bladder capasityLast max. bladder capasity (ml)	0.61 ± 0.140.85 ± 0.10(P = 0.004)	0.59 ± 0.14 1.10 ± 0.95 (P = 0.001)	0.60 ± 0.21 1.20 ± 0.11 (P = 0.001)	0.60 ± 0.18 1.01 ± 0.12 (P = 0.003)
Basal leak point pressureLast leak point pressure (cmH2O)	28.88 ± 5.5936.25 ± 4.27(P = 0.029)	29.50 ± 5.29 60.38 ± 6.14 (P = 0.001)	30.43 ± 4.24 69.57 ± 1.51 (P = 0.001)	30.86 ± 3.34 59.14 ± 5.21(P = 0.001)
Basal complianceLast compliance (ml/cmH2O)	0.021 ± 0.0020.023 ± 0.003(P = 0.044)	0.020 ± 0.002 0.018 ± 0.001 (P = 0.024)	0.019 ± 0.004 0.017 ± 0.002 (P = 0.045)	0.019 ± 0.0040.018 ± 0.002(P = 0.193)

**Figure 2:**
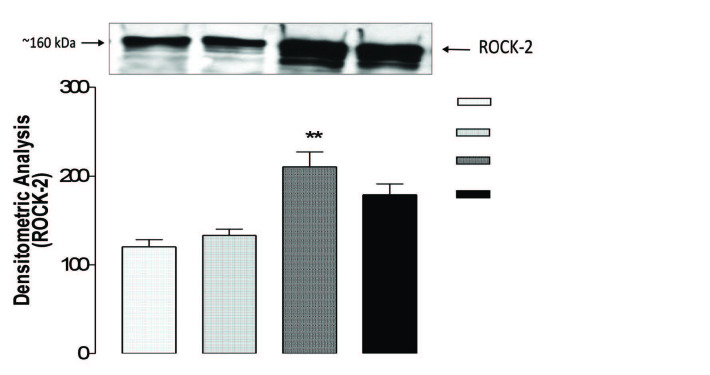
Rho kinase expression in all groups. ROCKII expression was measured by western-blotting. Density of bants was analyzed with Scion Image program. Data represent mean   standard errors of the mean (S.E.M.) of n observations. For statistical comparison, analysis of variance (ANOVA) followed by Bonferroni’s multiple comparison test was used. **P<0.01 (BOO versus BOO+orchiectomy).

As shown in Figure 2, ROCK-2 expression did not change significantly in the BOO group, compared with the sham group (P > 0.05). However, in the BOO + orchiectomy group, ROCK-2 expression significantly increased compared to the BOO group (P < 0.01). I.m. testosterone treatment added after BOO + orchiectomy failed to reverse the up-regulation of ROCK-2 observed in the BOO + orchiectomy group although ROCK-2 level tended to decrease. 

**Figure 3:**
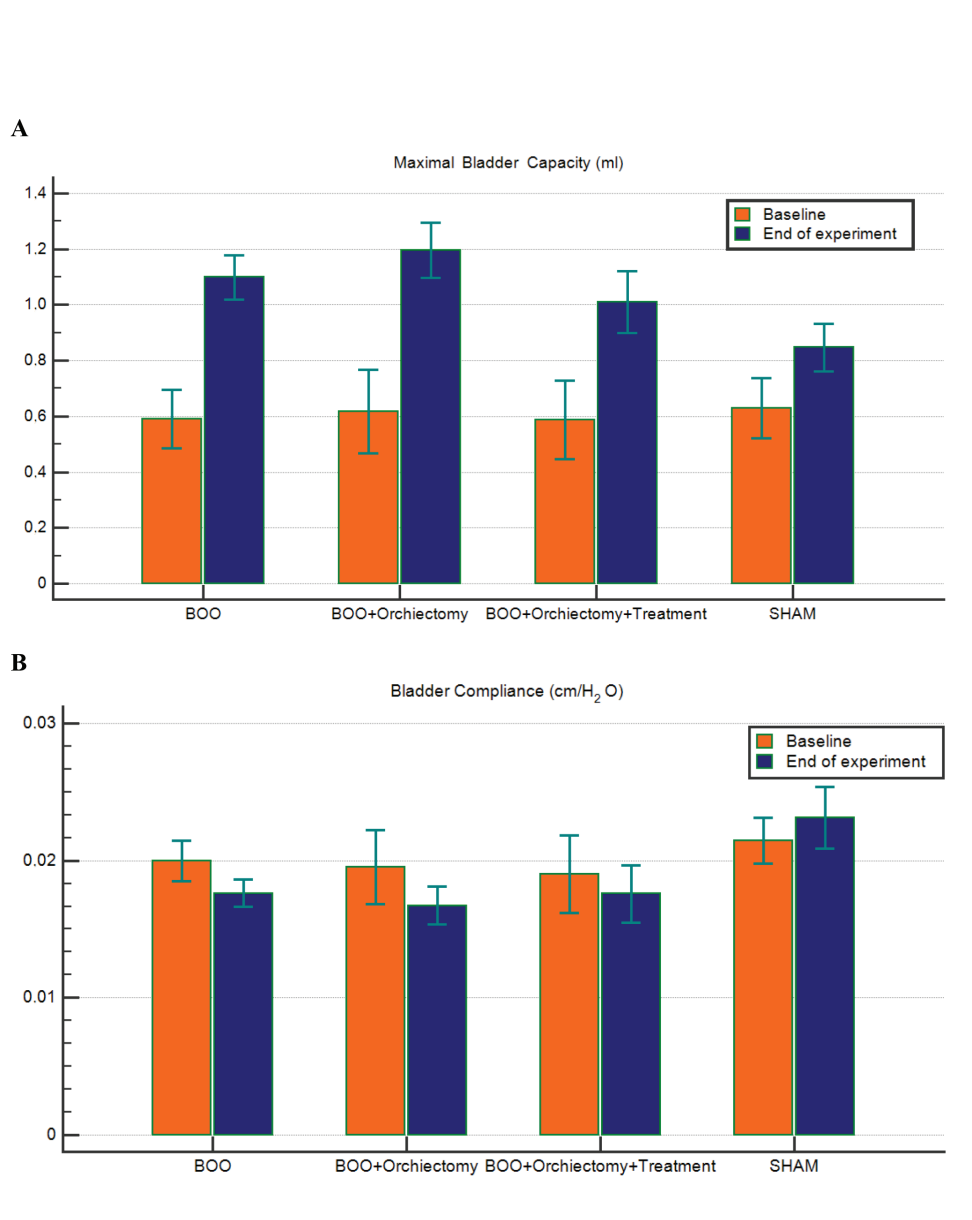
Mean maximal bladder capacity (A) and bladder compliance (B) from the beginning to the end of the experiment in all groups.

Figure 3 shows maximal bladder capacity and compliance from the beginning to the end of the experiment in all groups with the changes as percentages also as shown in Table 2. Changes in maximal bladder capacity and leak point pressure were higher in the BOO group than in the sham group (P = 0.026 and P = 0.001, respectively). Change in bladder compliance was lower in the BOO group, compared with the sham group (P = 0.002). However, the apoptosis index did not differ significantly between the two groups (P = 0.380). Changes in maximal bladder capacity and bladder compliance did not differ between the BOO + orchiectomy and BOO + orchiectomy + T treatment groups (P = 0.247 and P = 0.539, respectively). However, leak point pressure significantly more decreased in the BOO + orchiectomy + T treatment group than in the BOO + orchiectomy group (P = 0.045). In addition, the smooth muscle/collagen ratio was higher in the BOO + orchiectomy + T treatment group than in the BOO + orchiectomy group (P = 0.01). The total apoptosis index was lower in the BOO + orchiectomy + T treatment group than in the BOO + orchiectomy group, but the difference was not statistically significant (P = 0.181). 

**Table 2:** Changes as percentage in cystometric findings (maximal bladder capasity, leak point pressure and compliance) from the beginning to the end of the study and mean smooth muscle/collagen ratio and total apoptosis index at sacrification in all groups.

	Sham group (n = 8)	BOO group (n = 8)	BOO + orchiectomy group (n = 7)	BOO + orchiectomy + T treatment group (n = 7)
Change in maximal bladder capacity (%)	42.60	96.46	112.87	80.22
Change in leak point pressure (%)	29.26	110.65	131.68	94.78
Change in bladder Compliance (%)	9.10	–10.07	–11.67	–7.17
Smooth muscle/collagen ratio	2.80 ± 0.84	3.20 ± 0.76	1.90 ± 0.65	3.30 ± 0.67
Total apoptosis index	1.80 ± 1.30	3.24 ± 3.21	6.00 ± 3.16	3.60 ± 1.85

## 4. Discussion

In this study, we investigated effects of testosterone undecanoate therapy on the ROCK-2 expression and bladder behavior in the rat BOO and hypogonadism models. Smooth muscle system and function were essentially changed following BOO [3]. One of the main reasons that the bladder contraction function decreases is the inability to adapt to BOO [7]. That ROCK has a critical function in the organization of rat urinary bladder smooth muscle contraction and tone have been shown by most studies. ROCK enzymes are highly expressed in rat urinary bladder smooth muscle, and the selective ROCK inhibitor Y-27632 attenuates contractions [7]. Based on this, we analyzed differences in ROCK-2 expression following a model of BOO. Compared with the sham group, we came to the conclusion that ROCK-2 expression did not change meaningfully in the BOO group for two weeks. In a former study on the expression of ROCK isoforms in BOO rabbits, Bing et al. indicated that in mature male rabbits, ROCK α (ROCK-2) did not differ, supporting our data. However, in that study, ROCK β (ROCK-1) was overexpressed in the smooth muscle in response to BOO. 

Our research is different in terms of the time course of obstruction. Besides, the antibody usage and the level of obstruction performed may differ [2]. There is no data on the relationship between ageing and ROCK expression in the bladder tissue. Observations have shown that the structural proteins of the cytoskeleton, and its connection to the extracellular matrix with the plasma membrane are dynamically regulated and play an important role in contraction responses, as well as accompanying bladder contraction with cyclic ischemia/reperfusion (I/R). The severity of ischemia/reperfusion increases in the presence of increasing bladder thickness. Age related intolerance to cyclical I/R may cause activation of ROCK signaling [12]. In support, unilateral obstruction of rabbit ureter, the expression levels of ROCK-1 and ROCK-2 dramatically up-regulated, implicating that Rho/ROCK signaling pathway may be an important therapeutic target in for the treatment of particular urogenital diseases [13]. SLOH is associated with advancing age and characterized by typical symptoms and a deficiency in serum testosterone levels. Orchiectomized rats have been used as a model of testosterone deficiency due to hypogonadism [14]. However, SLOH is a condition characterized by a combination of central and peripheral hypogonadism. Surgical castration is a pure model of peripheral hypogonadism, and may not be an appropriate model of SLOH in human. In the present study, we performed BOO and bilateral orchiectomy, and then we investigated the effect of testosterone replacement therapy on bladder functions and smooth muscle/collagen content in orchiectomized mature male rats. 

Our experimental rat study showed that bladder capacity and smooth muscle/collagen content may decrease in the presence of testosterone deficiency. Androgens can stimulate stromal precursor cells differentiation into smooth muscle cells through the activation of androgen receptors. In their study, Lin et al. showed that the number of vessels, epithelial thickness, and muscle fibers increased in the bladder of rats receiving androgen/estrogen compared to the bladders of rats receiving isolated conjugated estrogen [15]. Few studies have reported that androgens also regulate growth and differentiation of vascular smooth muscle cells in bladder tissue [9]. Therefore, we investigated the effect of testosterone treatment on bladder functions and smooth muscle/collagen content in castrated adult rats. Androgen deprivation can interfere with smooth muscle differentiation. In this study, after performing orchiectomy we showed that bladder capacity and smooth muscle content decreased. Furthermore, following orchiectomy ROCK-2 expression increased significantly, but testosterone treatment failed to reverse this up-regulation although there was a tendency in ROCK-2 expression to decrease, which was not found to be significant. 

In our study, increases in maximum bladder capacity were significantly higher in the BOO group than in the sham group. In addition, the leak point pressure was higher in the BOO group than the sham group, and the difference was statistically significant. Bladder compliance was lower in the BOO group when compared with the sham group. Apoptotic indexes did not change in the sham group and in the BOO group. In the BOO + orchiectomy + T treatment group, maximal bladder capacity, and bladder compliance did not change. Leak point pressure decreased in the BOO + orchiectomy + T treatment group. In addition, smooth muscle/collagen ratio increased, and apoptosis indexes did not change in the bladder tissue.

A recent study of male rabbits showed that after testosterone injection, bladder capacity, and compliance increased blood testosterone levels in male rabbits undergoing bilateral orchiectomy [16]. These animal studies suggest that bladder dysfunction may be associated with androgen deficiency, and testosterone therapy can improve bladder function and smooth muscle/collagen ratio in orchiectomized animals.

Testosterone has been shown to cause occlusion with or without unstable detrusor contractions and may also cause changes in the voiding reflex. Maggi et al. treated adult male rats with daily injections of testosterone and measured bladder function [17]. They found that testosterone therapy may cause BOO, with increased residual urine volume and detrusor instability and without much change in bladder capacity. In another study by Pandita et al., testosterone treatment for 2 weeks increased prostate weight in adult male rats [18]. The enlargement of the prostate gland in response to androgen therapy can cause obstruction. However, in the present study, we used testosterone in the orchiectomized rats and normal range levels of testosterone achieved with testosterone therapy did not stimulate prostate growth to volumes greater than those measured during prior therapy. It has been reported that ROCK is involved in the pathophysiology of OAB symptoms induced by hypertension and diabetes [19].

Consequently, this study showed the relationship between testosterone and Rho-kinase-2 expression and hypogonadism rat model in BOO. ROCK-2 expression increased significantly after orchiectomy in bladder tissues, which was not significantly restored by testosterone replacement treatment. We suggest that, in hypogonadism, testosterone treatment has a substantial influence on BOO-induced detrusor overactivity without significantly change in ROCK-2 upregulation. Based on these results (e.g., up-regulation of ROCK-2 following bladder obstruction and orchiectomy), alternative treatment modalities might be considered, such as the use of ROCK inhibitors or/and testosterone replacement. However, further studies are needed to support these mechanisms related to testosterone treatment with ROCK inhibition in hypogonadism and its prevention of detrusor hypertrophy and detrusor overactivity.

## Informed consent

The experimental protocol was approved by the ethical committee on Animal Research at the University of Mersin School of Medicine (Approval No: 200917).

## References

[ref1] (2013). Effects of oral Rho kinase inhibitor fasudil on detrusor overactivity after bladder outlet obstruction in rats. Lower urinary tract symptoms.

[ref2] (2003). Obstruction-induced changes in urinary bladder smooth muscle contractility: a role for Rho kinase. American Journal of Physiology-Renal Physiology.

[ref3] (2004). Urinary bladder contraction and relaxation: physiology and pathophysiology. Physiological reviews.

[ref4] (2003). Regulation of force in vascular smooth muscle. Journal of molecular and cellular cardiology.

[ref5] (2000). Signal transduction by G-proteins, rho-kinase and protein phosphatase to smooth muscle and non-muscle myosin II. The Journal of Physiology.

[ref6] (2003). Expression of Rho-kinase and its functional role in the contractile activity of the mouse vas deferens. British journal of pharmacology.

[ref7] (2003). Expression and functional role of Rho-kinase in rat urinary bladder smooth muscle. British Journal of Pharmacology.

[ref8] (1997). Prostate size in hypogonadal men treated with a nonscrotal permeation-enhanced testosterone transdermal system. Urology.

[ref9] (2010). The effect of testosterone replacement therapy on bladder functions and histology in orchiectomized mature male rats. Urology.

[ref10] (2006). The effect of chronic inflammatory condition of the bladder and estrogen replacement therapy on bladder functions and histology in surgically menopause and chronic cystitis induced rats. Neurourology and Urodynamics.

[ref11] (2006). Contribution of Rho-kinase in human gallbladder contractions. European Journal of Pharmacology.

[ref12] (2001). The effect of bladder outflow obstruction on detrusor blood flow changes during the voiding cycle in conscious pigs. The Journal of Urology.

[ref13] (2007). Role of Rho-kinase in contractions of ureters from rabbits with unilateral ureteric obstruction. BJU International.

[ref14] (2000). Androgen deficiency induces high turnover osteopenia in aged male rats: a sequential histomorphometric study. Journal of Bone and Mineral Research.

[ref15] (2007). Effect of letrozole on urinary bladder function in the female rabbit. BJU International.

[ref16] (2003). Effects of different sex hormones on male rabbit urodynamics: an experimental study. Hormone Research.

[ref17] (1989). Infravesical outflow obstruction in rats: a comparison of two models. General Pharmacology.

[ref18] (1998). Testosterone‐induced prostatic growth in the rat causes bladder overactivity unrelated to detrusor hypertrophy. The Prostate.

[ref19] (2005). Rho‐kinase inhibition suppresses bladder hyperactivity in spontaneously hypertensive rats. Neurourology and Urodynamics.

